# A Review on 3D-Printed Miniaturized Devices for Point-of-Care-Testing Applications

**DOI:** 10.3390/bios15060340

**Published:** 2025-05-28

**Authors:** Amol S. Kulkarni, Sarika Khandelwal, Yogesh Thakre, Jyoti Rangole, Madhusudan B. Kulkarni, Manish Bhaiyya

**Affiliations:** 1 Department of Computer Science and Engineering, G. H. Raisoni University, Amravati 444701, MH, India; 2 Department of Computer Science and Engineering, G. H. Raisoni College of Engineering, Nagpur 440016, MH, India; 3 School of Computer Science and Engineering, Ramdeobaba University, Nagpur 440013, MH, India; 4 Department of Electronics and Telecommunication Engineering, Vidya Pratishthan’s Kamalnayan Bajaj Institute of Engineering & Technology, Baramati 413133, MH, India; 5 Department of Electronics and Communication Engineering, Manipal Institute of Technology, Manipal Academy of Higher Education (MAHE), Manipal 576104, KA, India; 6 Department of Chemical Engineering and the Russell Berrie Nanotechnology Institute, Technion-Israel Institute of Technology, Haifa 3200003, Israel

**Keywords:** 3D printing, precision healthcare, medical diagnosis, point-of-care testing, biosensors, additive manufacturing

## Abstract

Integrating three-dimensional printing (3DP) in healthcare has modernized medical diagnostics and therapies by presenting various accurate, efficient, and patient-specific tailored solutions. This review critically examines the integration of 3DP in the development of miniaturized devices specifically tailored for point-of-care testing (PoCT) applications in healthcare. Focusing on progressive additive manufacturing techniques, such as material extrusion, vat photopolymerization, and powder bed fusion, the review classifies and evaluates their contributions toward designing compact, portable, and patient-specific diagnostic devices. Unlike previous reviews that treat 3DP or PoCT generically, this work uniquely bridges the technical innovations of 3DP with clinical applications by analyzing wearable sensors, biosensors, lab-on-chip systems, and microfluidic platforms. It highlights recent case studies, performance metrics, and the role of 3DP in enhancing diagnostic speed, accessibility, and personalization. The review also explores challenges such as material standardization and regulatory hurdles while outlining future directions involving artificial intelligence (AI), the Internet of Things (IoT), and multifunctional integration. This focused assessment establishes 3DP as a transformative force in decentralized and precision healthcare.

## 1. Introduction

In recent times, 3DP, commonly known as additive manufacturing (AM), has promised to unlock new potential in many global industries. Three-dimensional printing, by the very nature of layer-on-layer fabrication processes, allows for creating very complex designs not usually manageable with traditional ways of manufacturing [1,2,3]. The initial scope of 3DP was limited to prototyping and design validation [4]. With time, it started expanding into newer areas like aerospace, automotive, fashion, and healthcare. Three-dimensional printing is pivotal in realizing these advanced processes of manufacturing because of its ability to manufacture complex geometries, allowing for lower material wastage and higher production cycles. The impact of three-dimensional printing is mostly felt in the healthcare sector, where it has not only improved the accuracy and customization of devices but also broadened the range of treatments offered to fit individual patient requirements [5,6,7]. From surgical models and implants to prosthetics and drug delivery systems, 3DP is transforming how medical professionals approach and treat a diagnosis. The ability to create patient-specific models and devices has made it easier to do everything from preoperative planning to the development of personalized medicine that improves clinical outcomes and increases patient satisfaction [8,9,10].

**Figure 1:**
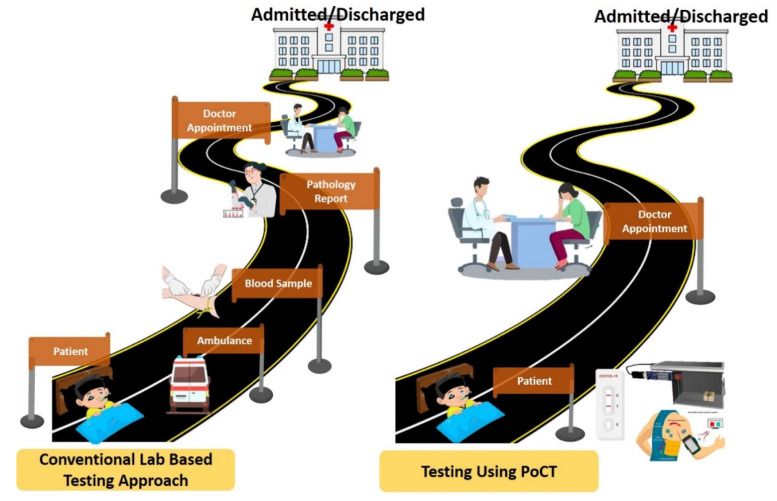
Comparison of conventional lab-based testing (**left**) and PoCT (**right**). PoCT offers faster diagnosis by reducing steps such as sample transport and centralized lab processing.

One of the most significant applications of 3DP in healthcare is PoCT. The left pathway depicted in Figure 1 shows the multiple steps involved in conventional lab-based testing, ambulance transport, sample collection, lab analysis, and reporting, resulting in delayed diagnosis. In contrast, the right pathway shows the streamlined PoCT process, where testing occurs near the patient with rapid results, thus significantly reducing the diagnostic turnaround time. PoCT includes tests which are used for diagnostics at or near where the patient is receiving treatment, allowing for quick results to be returned, with no need for central laboratories [11,12,13]. Diagnostic testing in the past took advantage of large laboratory infrastructure, which led to problems such as high costs, extended waiting periods, and a lack of trained personnel. Thus, PoCT devices have provided portable, inexpensive, and efficient solutions to the ever-increasing demand for rapid diagnostic testing [14,15,16]. With the development of 3DP technologies such as material extrusion, vat photopolymerization, powder bed fusion, and sheet lamination, the potential of PoCT devices has been further accentuated [17,18,19]. These technologies have made it possible to combine very compact, lightweight, and interactive custom-made diagnostic devices suitable for medical demands, such as infectious disease detection and chronic disease monitoring [8,20].

The integration of 3D printing into patient care concepts opens opportunities to decentralize healthcare, especially in resource-limited settings. The production of diagnostic devices on demand has rendered the possibility of extending diagnostics to distant and underdeveloped regions with limited access to ordinary laboratories [21,22]. For example, 3D printing enables the development of microfluidic devices and lab-on-chip systems, which optimize the ability to perform multiple diagnostic tests involving minimal sample volumes. Such developments have dramatically reduced costs attributed to diagnostic testing and ushered in possibilities for inclusive healthcare delivery [23,24]. Three-dimensionally printed PoCT devices may have a higher initial fabrication cost compared to paper-based diagnostics, but they offer competitive advantages in terms of multi-analyte detection, durability, reusability, and integration with digital technologies. Several studies have demonstrated that the per-unit cost of basic 3D-printed biosensors can range between USD 1 and 5, especially when using low-cost materials such as PLA or conductive filaments [11,25]. These devices are becoming increasingly suitable for resource-limited settings, particularly when factors like local fabrication, on-demand customization, and reduction in logistical costs are considered. Technological advancements within this framework are evidently interwoven with emerging fields such as artificial intelligence, machine learning, and the Internet of Things [26,27]. This synergy has led to the development of smart diagnostic devices that are able to analyze data in real time and remotely transmit results to healthcare providers. Wearable devices for PoCT equipped with biosensors and wireless communication functions are therefore increasingly entering the market. These devices allow for continuous monitoring of health and provide patients with valuable insights, enabling them to manage their conditions proactively [28,29,30]. These breakthroughs highlight the game-changing potential of 3D printing not only in disease diagnosis but also in enabling preventative healthcare.

Although several review articles have explored the applications of 3DP in diagnostics and healthcare, they are generally limited in scope and application. For example, Shakibania et al. provide a fabrication-focused overview of additive manufacturing techniques for diagnostic tools but do not deeply engage with PoCT-specific device integration or miniaturization [31]. Similarly, Chan et al. highlight early uses of 3D printing in microfluidic chip prototyping but lack a discussion of clinical performance or emerging domains like wearable sensors and AI-assisted analytics [11]. Han et al. [32] and Manzanares and Pumera [33] focus heavily on materials and analytical devices, offering useful insights into sensor construction but without a clear emphasis on system-level PoCT applications, end-user deployment, or digital integration. Other reviews, such as Su et al.’s [34], emphasize the functionalization of 3D-printed devices for chemical sensing, but these discussions remain largely within the domain of material chemistry and do not address broader diagnostic applicability. Likewise, Sharafeldin et al. [35] focus specifically on 3D-printed biosensor arrays, omitting the wider ecosystem of lab-on-chip systems, microfluidics, and real-world usability. Yang et al. [21] present a strong case for in vitro diagnostics using 3D printing, yet they overlook wearable formats, real-world PoCT deployment challenges, and integration with smart technologies like AI and IoT. In contrast, our review is novel in terms of both scope and structure. It brings together diverse classes of miniaturized diagnostic devices, wearable sensors, biosensors, lab-on-chip systems, and microfluidic platforms within a unified framework specifically centered on PoCT. We provide updated case studies from recent years (2020–2024) and assess device performance metrics such as cost, speed, and sensitivity. Furthermore, we discuss affordability, portability, and regulatory considerations, making this review both technologically rich and practically grounded. In doing so, we bridge a major gap in the current literature by providing a comprehensive, translationally relevant, and future-oriented perspective that supports researchers, clinicians, and developers alike. The structure of this review allows for a much more comprehensive understanding of 3DP technology and applications in PoCT by various audiences, starting from a discussion presenting the generic mechanisms of operation of the application of the various 3DP technologies, outlining their main features and applications in the healthcare sector, before further discussion of selected applications of 3DP in the PoCT realm like wearable devices, biosensors, lab-on-chip systems, and implantable devices. The obstacles to adopting 3DP in healthcare and the subsequent future research directions are also addressed here. Based on the latest advances, this article attempts to articulate some case studies to create an information tool for those engaged in research, practice, and business around 3DP and innovations in health.

## 2. Three-Dimensional Printing Technology Classification

**Figure 2:**
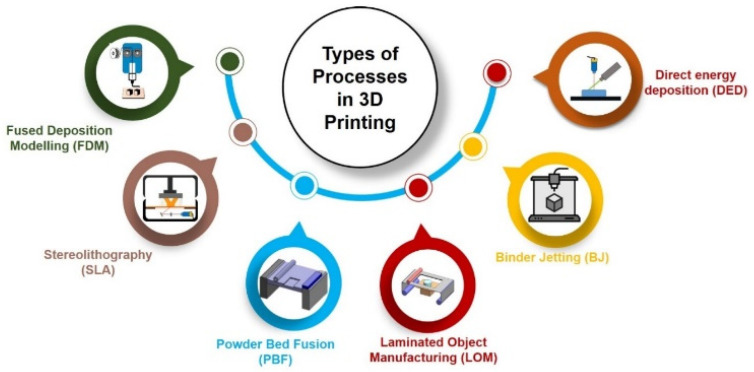
Schematic illustration of types of processes in 3DP.

AM has recently advanced quickly, primarily due to the demand for fast prototyping. Since certain AM technologies are better suited for specific applications, various technologies have been created that accept multiple materials to produce complex models of high quality and with a high resolution. These processes are broken down into three additive manufacturing categories [36,37]. The first is a sintering technique, whereby the material is heated to a level that leads to the prototype’s detailed features but the material does not melt. The second is melting, which exposes the material to a powerful energy beam (typically a laser beam or an electron beam) so that the material is completely melted. Finally, stereolithography relies on a liquid photopolymerization system to produce a prototype layer by layer. The following sections will examine how each of these additive manufacturing technologies works with illustrations appropriate to each category. Figure 2 illustrates the different classifications of 3D printing technologies.

### 2.1. Material Extrusion/Fused Deposition Modelling (FDM)

**Figure 3:**
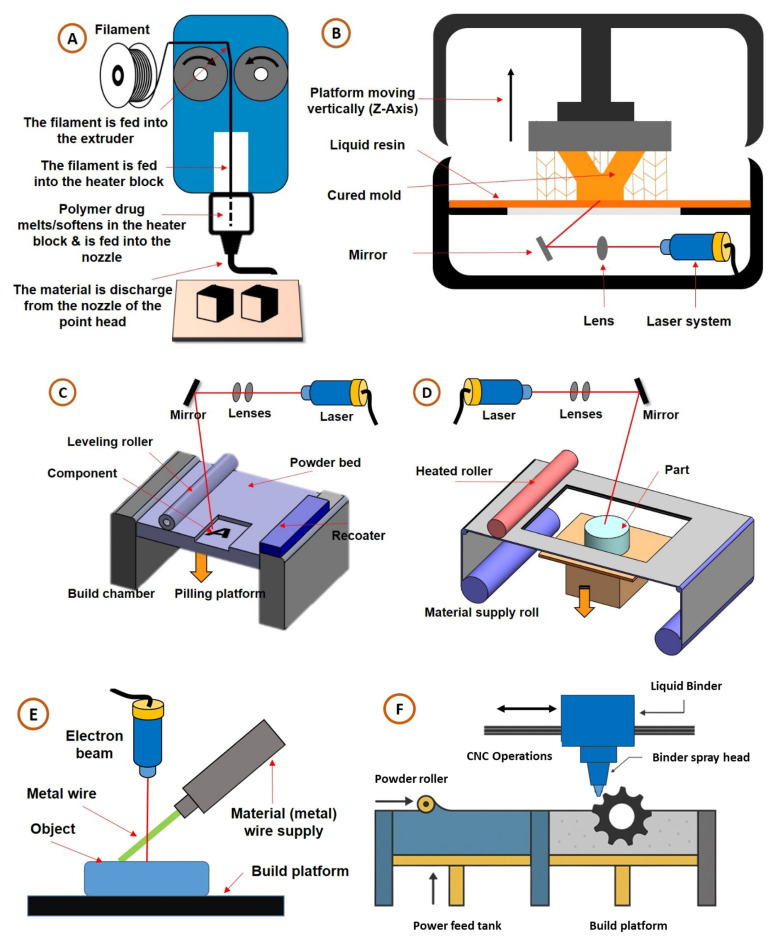
Illustration of the various methods that can be used in 3D printing: (**A**) the Fused Deposition Modelling (FDM) method, where melted plastic is deposited layer by layer to build objects; (**B**) the stereolithography (SLA) method, which uses light to cure the liquid resin layer by layer; (**C**) the powder bed fusion (PBF) method, where a laser or electron beam fuses powdered material to create solid parts; (**D**) the laminated objection manufacturing (LOM) method, which builds and cuts layers of material to create the object; (**E**) the direct energy deposition (DED) method, which uses focused energy to melt the material as it is added to build up an object; (**F**) Binder Jetting (BJ) refers to a 3D printing technique that produces items by depositing a liquid binder in a powder bed.

FDM is an additive process that builds 3D objects from a thermoplastic filament in, well, layers. It is quite straightforward: there is filament feeding, melting and extrusion, and layer-by-layer building, as can be seen in Figure 3A. This technology starts with a 3D printer loaded with a spool of a thermoplastic filament, usually PLA or ABS. The filament acts as the printer’s feedstock. More importantly, this makes for a pragmatic and economically wise solution for users. The filament is heated until molten and is then extruded through the printer nozzle onto the build plate or upon layers previously deposited in a pattern defined precisely through the designed specifications. Layer-by-layer deposition is the basis of FDM technology, whereby the nozzle moves in a controlled manner, depositing successive layers of melted material. After each layer is deposited, this material hardens at an environmental temperature over time, gradually forming into the physical representation of the object in question. This process is repeated until the desired object is achieved, with the user being advised to select a different layer resolution based on needs [1,38,39].

### 2.2. Vat Photopolymerization/Stereolithography (SLA)

SLA is a light-based 3D printing technology that makes use of the photopolymerization process to create complexly shaped objects with high precision and detail. A liquid photopolymer resin that goes through a phase transformation into UV (ultraviolet) light is used. A starting liquid resin of photopolymers has to be one that, upon exposure, is suited for chemical transformation into any one of the desired states through optical curing of allowed polymerizations. Upon exposure to UV light, the resin undergoes polymerization, a chemical reaction whereby molecules in the resin are linked together to form chains, allowing for the transformation of the resin from a liquid to a solid. This selective solidification is performed by computer-controlled systems that progressively apply UV light, thus determining which areas of the resin solidify. Wherever the light shines, it solidifies the resin, progressively forming the object under printing layer by layer by selectively solidifying any successive layer of resin with the ultraviolet light. The working concept of SLA printing is illustrated in Figure 3B [39].

SLA is gaining wide acceptance in healthcare because of its precision and capability of building complex models. This includes the making of high-resolution anatomical models for surgical planning. These models help a surgeon visualize a complex procedure before performing it on a patient [40]. Moreover, biocompatible resins compatible with SLA clear the platform for fabricating dental crowns, aligners, and hearing aids. SLA would be expected to function ten times more efficiently in crafting these patient-specific devices thanks to its ability to readily produce smooth surface finishes and geometries with high accuracy. Continuous developments seen in SLA have contributed to an expanded scope in prosthetics and orthotics, providing specific solutions to enhance patient comfort and function [41]. Beyond medical models and devices, SLA has shown a huge potential in drug delivery and tissue engineering [42]. However, despite its fine resolution and precision, SLA has limitations of slow build speeds and material constraints compared to powder bed fusion (PBF). Despite the limitations, research is in progress to improve upon these weaknesses. For example, the new application development has resulted in enhancements in photosensitive resin formulations and faster curing technologies, making SLA more versatile and efficient in scope within the healthcare industry. As SLA matures further, the coupling of this technology with multiple-material printing and digital light processing offers a window into furthering its applications for multifunctional devices, including combined biosensors and implants, with the potential to augment patient care in the coming years [43].

### 2.3. Powder Bed Fusion (PBF)

In PBF, powder materials are used to make products layer by layer. In the intricate workflow of PBF, there are several grossly different stages that contribute to the accurate making of sophisticated structures. This digital preparation starts with the detailed design of the desired object in special software. The 3D model for a defined object is sliced into thin cross-sectional layers, which allows all further fabrication steps. Then, in the preparation of the material, a fine bed of powdered material is spread uniformly over the build platform, with relevant materials ranging from metals and polymers to ceramics. This particulate bed acts as the canvas in building the final object. The layer application is essential, which involves selectively fusing or melting a thin layer of the powdered material with a heat source like a laser or electron beam, as shown in Figure 3C. This fusion of the powdered material according to the slicing of the 3D model pattern of the first layer is intricately circumscribed. Then, after the first layer’s compaction, another thin layer of powdered material is delicately spread on top of it; thus, this two-step process goes on extensively, layer by layer, connecting each layer specifically with selective fusion to the one beneath it according to the pattern outlined in the digital model. The repeat process takes place until the complete object is built, with the digital model acting as a guiding blueprint throughout the fabrication process. Lastly, cooling and solidification are ensured, with the layers deposited cooled and sealed together such that full structural status is achieved with the completed product [1,44].

The capability of PBF to produce elaborately detailed, biocompatible structures has set in motion the world of personalized medicine, incorporating implants and drug delivery systems. Below, we will explore advancements and applications of PBF in healthcare driven by a substantial body of recent studies and innovations. Abdalla et al. (2024) advanced the idea of PBF as a vehicle for providing adapted solutions to personalized healthcare, particularly implant and drug delivery systems [45]. Hussain et al., in 2024, accentuated PBF’s versatility in multilateral elaboration, which is critical for developing advanced bio-fabrication and multifunctional healthcare devices [46]. PBF’s ability to create biocompatible scaffolding has been well studied. Li et al., in their 2020 study, used WE43 Mg alloys made by PBF as implants and illustrated their biocompatibility and critical mechanical properties relating to medical promulgations [47]. Along the same lines, McGregor et al., in 2021, examined architectural lattice designs with integration into bone, reaffirming PBF’s potential in orthopedic applications [48]. Apart from healthcare products, PBF has been a flagship technology in material innovation. For instance, Schäfer et al., in 2023, developed magneto-active composites with locally adapted stiffness, which can be employed in a variety of biomedical applications [49]. Verifying the selection of appropriate fabrication methods for specific healthcare needs was another aspect that Nick et al. considered in 2022 in an evaluation of active pharmacological biology [50]. Moreover, the precision and versatility of PBF extend into the pharmaceutical field. Trenfield et al., in 2018, and Awad et al., in 2021, showcased their production of customized drug delivery systems and dosage forms, which significantly improved therapeutic outcomes [51,52]. These studies substantiate PBF’s potential to take on complex healthcare problems through innovative additive manufacturing techniques. Islam et al., in 2021, explored biomimetic design strategies, furthering the argument of PBF regarding the similarities in the replication of structurally complex biological structures [53]. Lastly, Dizon et al., in 2018, elaborated on the mechanical properties of polymers used within PBF for insight into material selection regarding healthcare applications [54]. All the above signify advancements in the use of PBF to boost healthcare productively, developing precise, robust, and biocompatible solutions.

### 2.4. Sheet Lamination/Laminated Object Manufacturing (LOM)

LOM is a 3DP process based on a layering technique that builds objects in three dimensions. It begins with the stacking of thin sheets of material, which can comprise paper, plastic, or metal, and which are incorporated into the body of the object created [1,55]. A computer-controlled laser or blade then cuts every layer into shape according to a pre-designed 3D model. The accuracy with which this is performed allows for the faithful reproduction of the most complicated geometries and designs. The superfluous material is removed after the layer is cut to reveal the layer of the shape. One of the critical moments in the LOM process is the bonding of the layers, which can be performed via heat, pressure, or adhesives, depending on the material. This method of bonding creates a dense, physical, and continuous structure of consolidated layers. Unlike other additive manufacturing techniques, LOM does not involve melting or fusing the material, rendering it a relatively low-heat and low-energy-consuming method [56]. This not only reduces operational costs but also makes the technique compatible with materials that may degrade at high temperatures. The working concept of LOM printing is shown in Figure 3D. One of the great benefits of LOM is its wide range of usage, since the material choice fits applications in industries from packaging to healthcare. In the medical field, Chia and Wu et al. discussed the applications of LOM in the fabrication of anatomical models for surgical planning, prosthetics, and orthotics to improve accuracy in planned surgeries for personalized options for patients [57]. Advanced applications of LOM comprise lab-on-a-chip devices for point-of-care diagnostics. Comina et al. [58] pointed out its accuracy in creating fine microfluidic channels that are crucial for portable diagnostic systems. An opportunity has been found for bio-inspired laminates that mimic the properties of natural tissue in regenerative medicine, as noted by Murphy and Atala [59]. These advantages are somewhat restricted, since LOM has limits, such as its microscopic resolution and strong bonding capabilities, in comparison to powder bed fusion (PBF). However, it has been pointed out through ongoing research that the problems with LOM can still be improved by concentrating on laminated adhesive properties while investigating new materials for lamination. Advances in biocompatible adhesives and laser technologies are stacking the odds for LOM to realize greater accuracy and further its reach in healthcare.

### 2.5. Binder Jetting (BJ)

BJ refers to a 3D printing technique that produces items by depositing a liquid binder in a powder bed, as shown in Figure 3F. The printing process begins by distributing a thin layer of powder, which may consist of metal, ceramic, or plastic, across a platform in a uniform manner. The print head then travels across the surface of the layer and “prints” by depositing small amounts of liquid binder in the shape of the part. This process continues layer by layer. Each layer is built on top of the previously formed layer, and the process continues until the completed part is formed. The completed print usually undergoes post-processing, such as heating or curing, to strengthen and enhance durability [1].

### 2.6. Direct Energy Deposition (DED)

A DED process is a 3DP process that increases a material layer by layer, using a directed heat source (e.g., laser or electron beam), as shown in Figure 3E. The process begins by depositing material (usually powder or wire) onto a surface or an existing layer. The energy source then melts the material and creates a frozen layer. This continues until the full, solid, three-dimensional object is created directly from a digital design. The layering can be applied to create complex features and fit any design specifications. Furthermore, DED systems can deliver real-time monitoring and control analysis that ensures precision for the whole printing operation [38]. For an easy understanding of the key features and capabilities of each 3D printing technology, Table 1 compares them based on some important parameters, namely, materials used, resolution, speed, cost, energy efficiency, surface finish, and strength of output. The detailed comparative table shows both the capabilities and weaknesses of every method under discussion, the latter limiting their use in health science and beyond. In-depth comparisons can help enable proper selections in choosing a 3D printing method that meets requirements efficiently.

### 2.7. Material Considerations in 3DP PoCT Devices

The performance and dependability of 3D-printed PoCT devices depend on the selection of materials. In addition to establishing structural and mechanical integrity, materials govern the sensing performance, biocompatibility, conductivity, and wearability of diagnostic devices. Each new material introduced in additive manufacturing pushes the frontier forward and represents an opportunity to move away from laboratory-based diagnostics to personal, portable healthcare systems [62].

#### 2.7.1. Thermoplastics and Conductive Composites

Polylactic acid (PLA) is a biodegradable thermoplastic that is frequently used, especially in FDM, because of its ease of printing and biocompatibility. Even though PLA has inherent electrical insulating properties, it can be modified for electrochemical applications. The incorporation of conductive fillers such as graphene or carbon black into PLA matrices has provided materials that can be used for conductive filaments, specifically to create suitable biosensing materials. Graphene PLA Composites: These composites have displayed improved electrochemical properties that make them well-suited to detecting biomarkers such as dopamine and uric acid. Furthermore, post-processing treatments (solvent and electrochemical activation) can improve the sensitivity and reproducibility of a composite. Commercial Conductive Filaments: Products like Black Magic^®^ (ProtoPlant, Burbank, CA, USA) (PLA with ~8 wt % graphene) and Proto-Pasta^®^ (ProtoPlant, Vancouver, WA, USA) (PLA with ~21 wt % carbon black) have been employed to fabricate 3D-printed sensors, offering a balance between printability and conductivity [63,64,65].

#### 2.7.2. Photopolymer Resins for Microfluidic Devices

Photopolymer resins used in SLA and digital light processing processes have high-resolution capabilities that are beneficial in creating microfluidic PoCT devices. The type of resin selected is important because it determines the resulting optical clarity, mechanical strength, and biocompatibility. Biocompatible Resins: The resins described in the literature include some types of UV absorbers, such as avobenzone, which lead to the development of high-resolution microfluidic devices that are suitable for biological use. Bio-Med Clear Resin (Formlabs Inc., Somerville, MA, USA): This photopolymer resin is clear and biocompatible after processing. Therefore, it is an appropriate candidate for medical devices that require visualization and accuracy [66,67].

#### 2.7.3. Elastomers for Flexible and Wearable Sensors

Flexible materials allow for conformal wearable PoCT devices that stay in good contact with a dynamically moving surface of the body. Elastomeric materials like polydimethyl siloxane (PDMS) and styrene–ethylene–butadiene–styrene (SEBS) are preferred for their mechanical properties and skin compatibility. PDMS: PDMS is commonly used in microfluidic devices because it allows for optical transparency and flexibility. Additionally, the curing of PDMS could be inhibited by the 3D-printed mold that is a part of the rapid prototyping phase, requiring additional surface treatment or a different polymer. SEBS: SEBS is a thermoplastic elastomer with elasticity and biocompatibility, making it particularly useful for wearable sensors that require stretchability and comfort, regardless of the challenges associated with working with an elastomeric surface [68,69].

#### 2.7.4. Advanced Functional Materials

Hydrogels, MXenes, and metal–organic frameworks (MOFs) are new materials that are being researched for their special properties in PoCT. Hydrogels: These materials have a high water content and biocompatibility [70]. This means that they are a good, suitable alternative for biosensors that mimic biological tissues. MXenes: MXenes are two-dimensional materials with high electrical conductivity. Their sensitivity and high flexibility enable researchers to develop them as biosensors in wearable devices. Like GO, there are various composite MXene materials that can potentially be utilized in biosensors. Metal–Organic Frameworks (MOFs): MOFs have a high surface area and porosity, making them beneficial for use in biosensors, as they enhance sensitivity and selectivity to detect and quantify various analytes [71]. Table 2 shows the material properties and their impact on 3D-printed PoCT devices.

## 3. Applications of 3D Printing Technology

Technological progress in 3DP has provided a few opportunities in multiple domains, altering how products are engineered and manufactured. This section highlights the distinct applications of 3DP technologies, including wearable and implantable devices, highly advanced biosensors, and lab-on-chip devices.

### 3.1. Three-Dimensionally Printed Wearable Devices for PoCT Applications

The convergence of 3D printing technology with wearable devices has changed healthcare, particularly in biosensing applications. With advancements in customization, precision, and flexibility, 3DP enables the fabrication of wearable sensors tailored to individual users, facilitating real-time health monitoring and diagnostics. Unlike conventional methods, these printed sensors integrate soft materials, nanocomposites, and biomaterials, allowing for improved biocompatibility, stretchability, and sensitivity. The following section explores several case studies that exemplify these innovations, highlighting their significance in modern medical applications.

**Figure 4:**
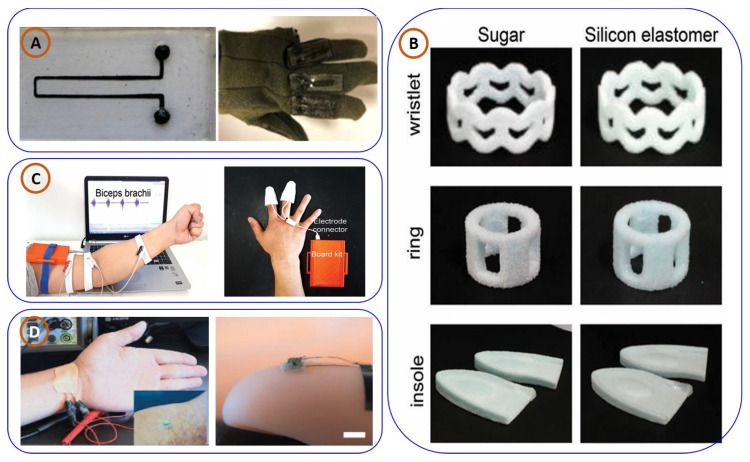
Illustrations of various 3D-printed (3DP) wearable sensor technologies designed for health monitoring and flexible electronics: (**A**) a piezoresistive flexible strain sensor using multi-walled carbon nanotubes (MWNTs) for wearable sensing applications, adapted from [72], with permission from Springer; (**B**) a wearable biosensor developed with soft 3D-printed materials for personalized healthcare monitoring, adapted from [74], with the permission of Wiley; (**C**) a stretchable tactile sensor created through 3D printing for monitoring pulse and finger movements, ideal for wearable electronics, adapted from [73], with the permission of Wiley; (**D**) a capacitive sensor produced using 3DP for wearable technology applications, adapted from [75], with the permission of the Royal Society of Chemistry.

Abshirin Mohammad et al. [72] developed 3D-printed flexible strain sensors using a nanocomposite of multi-walled carbon nanotubes and PDMS (Figure 4A). These piezoresistive sensors, integrated into a wearable glove, demonstrated effective real-time motion tracking for rehabilitation and assistive technologies. Complementing this, Dong Hae Ho and his team [1] engineered custom-fit 3D-printed wearable biosensors using soft-material printing (Figure 4B). Their devices captured diverse biosignals, EMG, EEG, EDA, and body motion, with high signal-to-noise ratios, showing promise in personalized healthcare and diagnostics. Further advancing this field, Shuang-Zhuang Guo et al. [73] fabricated stretchable tactile sensors via ultrathin-film patterning and stretchable polymer composites (Figure 4C). These sensors distinguished nuanced human motions, making them suitable for prosthetics and rehabilitation applications.

**Figure 5:**
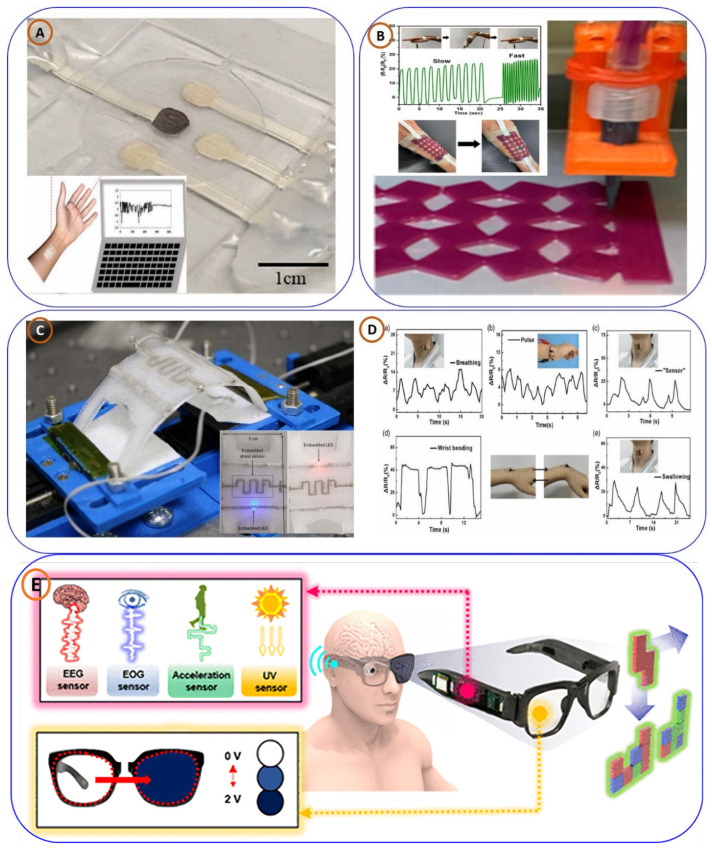
(**A**) A 3D-printed wearable patch for continuous health monitoring through sweat analysis, taken from [76], with the permission of Wiley. (**B**) A 3D-printed platform for colorimetric and mechanical sensing, designed for continuous health monitoring applications, taken from [77], copyright American Chemical Society. (**C**) A 3D-printed wearable sensor for monitoring knee joint movement, taken from [78], copyright IEEE. (**D**) Flexible piezoresistive sensors for detecting throat activities such as swallowing, speaking, and breathing [79] (**a**–**e**), with the permission of Wiley. (**E**) Three-dimensionally printed eyeglasses capable of detecting body movements and posture changes, taken from [80], with the permission of ACS.

Expanding the capabilities of materials for wearable sensors was achieved by Xiang-Yu Yin and colleagues [75], who introduced transparent, stretchable capacitive sensors using ionically conductive hydrogels (Figure 4D). Their work highlighted the role of 3D printing in enabling complex architectures for high-sensitivity physiological monitoring and human–machine interfaces. In biochemical sensing, Taeil Kim et al. [76] presented a 3D-printed wearable patch (Figure 5A) for sweat-based continuous health monitoring. Integrated microfluidics enabled accurate measurement of electrolytes (Na^+^, K^+^, and Ca^2+^), validated against standard lab tools. Lastly, Souvik Pal and co-authors [77] developed a multifunctional MOF-based ion gel sensor (Figure 5B) for combined colorimetric and mechanical sensing. The use of deep eutectic solvents and auxetic structures enhanced its durability, suggesting broad applicability in smart textiles and wearable electronics. Habib Nassar et al. [78] demonstrated the integration of smart sensing structures into wearable devices via 3D printing (Figure 5C). By embedding strain sensors and electronic components within polymeric materials using a silver–palladium metallic paste extruder, they developed a motion sensor specifically tailored for knee joint monitoring, highlighting its relevance for healthcare applications. Building on piezoresistive sensing, Lijun Mat [79] fabricated a flexible sensor using SEBS infused with carbon nanotubes via FDM (Figure 5D). The device showed high flexibility, sensitivity, and rapid response, proving effective for tracking human activities like swallowing, speaking, and breathing.

Integrating wearable electronics with human–machine interaction, a team led by Joong Hoon Lee [14] developed novel 3D-printed eyeglasses (shown in Figure 5E) that use embedded sensors and electrodes to record various signals related to a person’s biology and environment. The eyeglasses are made using flexible and stretchable carbon nanotube/polydimethylsiloxane (CNT/PDMS) electrodes to capture electrophysiological signals, for instance, electroencephalograms (EEG) and electrooculograms (EOG). The research encompasses many applications, such as detecting and recording body movement and posture, detection of UV exposure, interaction with digital systems, and more. Such results could aid in the development of digital healthcare, virtual reality (VR), and augmented reality (AR) [80]. In another biocompatible approach, Sahar et al. developed a wearable, bendable, and 3D-printed sensor for continuous, real-time pH monitoring, with no battery required to operate it, which has demonstrated very good sensitivity (51.76 mV/pH) and selectivity and showed stability across a broad pH range (3.0–10.0). The device also showed excellent mechanical flexibility and a 90% cell survival rate, reflecting good biocompatibility. This potentially indicates future applications in non-invasive healthcare monitoring and next-generation wearable technologies [81].

The aforementioned studies illustrate the remarkable diversity and adaptability of 3D-printed wearable sensors in healthcare and PoCT applications. As 3DP continues to evolve, its applications in biosensing, rehabilitation, and real-time diagnostics will further drive personalized healthcare solutions. To summarize these advancements, the following Table 3 presents a comparative overview of various 3D-printed wearable sensors, categorizing them by their application, material composition, sensing mechanism, and key performance metrics.

### 3.2. Three-Dimensionally Printed Biosensors for PoCT Applications

Three-dimensional printing technology has transformed the progress of biosensors in the fabrication of miniaturized, portable, low-cost, sensitive, and highly selective devices suitable for PoCT. This development is significantly impacting global healthcare, particularly in terms of improving access and facilitating earlier diagnosis. This section explores the latest developments and research that highlight how 3DP has transformed biosensor fabrication using electrochemical (EC), electrochemiluminescence (ECL), and chemiluminescence (CL) sensing approaches. In this regard, these new applications show that 3DP is pioneering the next generation of smart, efficient, and patient-friendly diagnostic tools for PoCT.

#### 3.2.1. Three-Dimensionally Printed Electrochemical Sensors for PoCT Applications

Electrochemical sensing is a powerful analytical technique that quantifies a broad range of analytes, including biomolecules, pathogens, and chemicals, by detecting electrical signals generated during redox reactions [91]. Its inherent high sensitivity, rapid response, and adaptability make it invaluable in healthcare, environmental monitoring, and food safety. Electrochemical sensors operate by measuring changes in current or potential at the electrode’s surface, which reflect the chemical state of analytes in solution. Modern electrochemical techniques, such as amperometry, voltammetry, and impedance spectroscopy, allow tailored detection approaches for diverse analytical needs [92,93]. Recent advances in 3D printing have significantly enhanced electrochemical sensor design and performance. Compared to conventional fabrication, 3D-printed sensors, particularly those made with conductive materials like graphene-infused PLA, offer improved sensitivity, lower detection limits, and faster response times due to precisely engineered microstructures that boost electron transfer kinetics. Furthermore, the digital fabrication process ensures high reproducibility and minimizes batch variability, making these sensors ideal for high-throughput, reliable use in clinical diagnostics and environmental applications [94,95].

**Figure 6:**
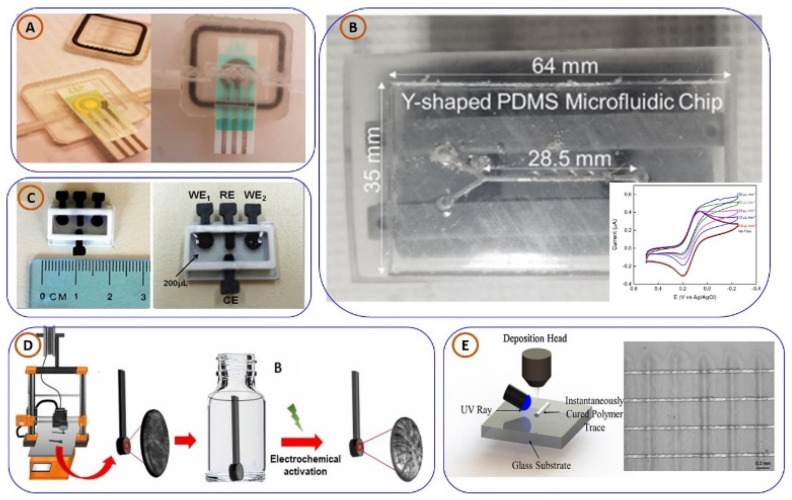
Various innovative 3DP electrochemical sensing devices: (**A**) a 3D-printed continuous flow system using MWCNT electrodes, adapted from [96], copyright MDPI; (**B**) a microfluidic device created via 3D printing for the simultaneous detection of insulin and ATP, adapted from [97], copyright Elsevier; (**C**) a 3D-printed chip designed to detect cholesterol and choline at the same time, adapted from [98]; (**D**) a conductive filament-based electrode developed using 3D printing for detecting hydrogen peroxide, adapted from [99], copyright Elsevier; and (**E**) silver microelectrode arrays, fabricated through 3D printing, used for detecting both hydrogen peroxide and glucose, adapted from [100], copyright Elsevier.

One of the first approaches in this domain involved the use of multi-walled carbon nanotubes (MWCNTs) for manufacturing high-performance electrochemical biosensors. Agitating this work towards further sensitivity, Samar Damiatia et al. [96] presented a novel study for a flow system printed in 3D based on MWCNT electrode principles. These electrodes have been successfully applied for the detection of hepatic oval cells, as shown in Figure 6A. A scaffold of a chitosan film into which oval cell marker antibodies (anti-OV6-Ab) are embedded has been fabricated using the screen-printing methodology. Thin-film electrodes show unique MWCNT properties, such as large surface areas and efficient immobilization, leading to an increase in overall electrochemical sensor sensitivity [78]. Furthermore, in the research on advancing electrochemical detection, microfluidic systems are incorporated with 3DP, as shown in work by Israel’s Belmonte et al. Rapid prototyping of epoxy-embedded electrodes and microfluidic devices through 3D printing was developed for use in electrochemical detection under flow conditions, as depicted in Figure 6B. The authors’ work emphasizes the new opportunities offered by 3D printing for the rapid production of molds and illustrates the integration of an electrochemical aptamer-based (E-AB) sensor specific to ATP into microfluidic devices [97].

The detection of multiple biomarkers simultaneously is an essential feature of advanced biosensing applications. Eleni Koukouviti and her group [98] developed a 3D-printed enzymatic microchip that can be used for multiplexed electrochemical biosensing. The microchip, illustrated in Figure 6C, can be quickly and accurately used to screen for disease. It demonstrated dual detection of relevant cardiac biomarkers, cholesterol and choline, indicating the potential for routine health monitoring and early diagnosis of disease. In a separate study, Adaris M. Lopez Marzo et al. [99] demonstrated the fabrication of conductive electrodes using 3DP, which were modified with horseradish peroxidase (HRP) for the electrochemical detection of hydrogen peroxide (Figure 6D). Their research discusses the biocompatibility of the 3D-printed graphene electrodes and their importance in the field of third-generation biosensors. Third-generation biosensors operate without an external electron mediator, simplifying the design and increasing sensitivity.

**Figure 7:**
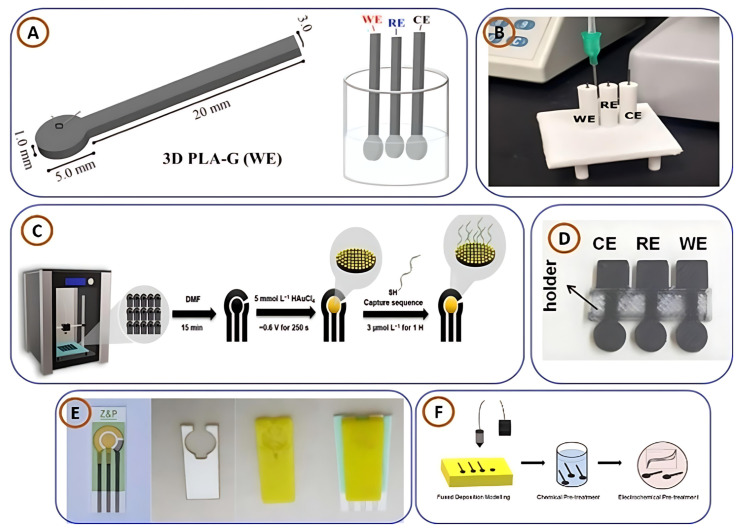
Figure 8 illustrates various innovative applications of 3D-printed (3DP) sensors and electrodes: (**A**) a 3D-printed conductive filament-based electrode used to detect L-methionine [101], copyright Elsevier; (**B**) 3D-printed biosensors designed for detecting harmful pathogens [102], copyright MDPI; (**C**) a 3DP three-electrode device capable of detecting both SARS-CoV-2 and creatinine [103], copyright MDPI; (**D**) a 3DP sensor developed for identifying caffeine and mercury [104], copyright Elsevier; (**E**) 3D-printed electrodes used for detecting the CD133 marker, important in cancer research [25]; and (**F**) a 3DP electrode designed to detect mycotoxins in food, ensuring safety and quality [105], copyright Elsevier.

The growing customizability of microelectrode arrays (MEAs) has led to more precise analyte detection. Haipeng Yang et al. [100] developed a 3DP silver MEA using an aerosol jet-based direct-write process (Figure 6E), offering adjustable electrode spacing for flexible circuit design. These MEAs are not only low-cost and environmentally friendly but also demonstrate effective detection of hydrogen peroxide and glucose, with a detection limit as low as 1.75 µM for glucose. In another study, Cristiane Kalinke and colleagues [101] designed a 3D-printed biosensor for L-methionine detection using PLA-graphene (PLA-G) filaments (Figure 7A). The sensor, suitable for miniaturization and customization, achieved a wide linear range (5–3000 µM) with a detection limit of 1.39 µM. Its practical applicability was validated through spiked human plasma samples. Expanding into pathogen detection, Samir Malhotra et al. [102] created a rapid and low-cost 3D-printed biosensor for Escherichia coli (Figure 7B), utilizing a modified pencil electrode. The sensor detected E. coli within 15 min, with detection and quantification limits of 53 CFU/mL and 270 CFU/mL, respectively. Its speed, portability, and potential for wireless integration make it a strong candidate for PoCT and biosafety applications. In response to the international observation of the pandemic, Silva et al. [103] developed a low-cost, disposable 3D-printed electrode modified with gold particles to detect both SARS-CoV-2 and creatinine (Figure 7C). The measurements provided by this biosensor are impressive in terms of the distribution, rapid hybridization time and sensitivity, potential for point-of-care real-world applications, and low limits of detection. The biological material immobilized directly onto the 3D-printed electrode offers a reliable, efficient, and rapid method for the detection of SARS-CoV-2 viruses during times of health distress. In addition, the device exhibited a detection limit of 0.30 µM for complementary DNA (cDNA), signaling that the device could be used for point-of-care testing during times of health distress.

**Figure 8:**
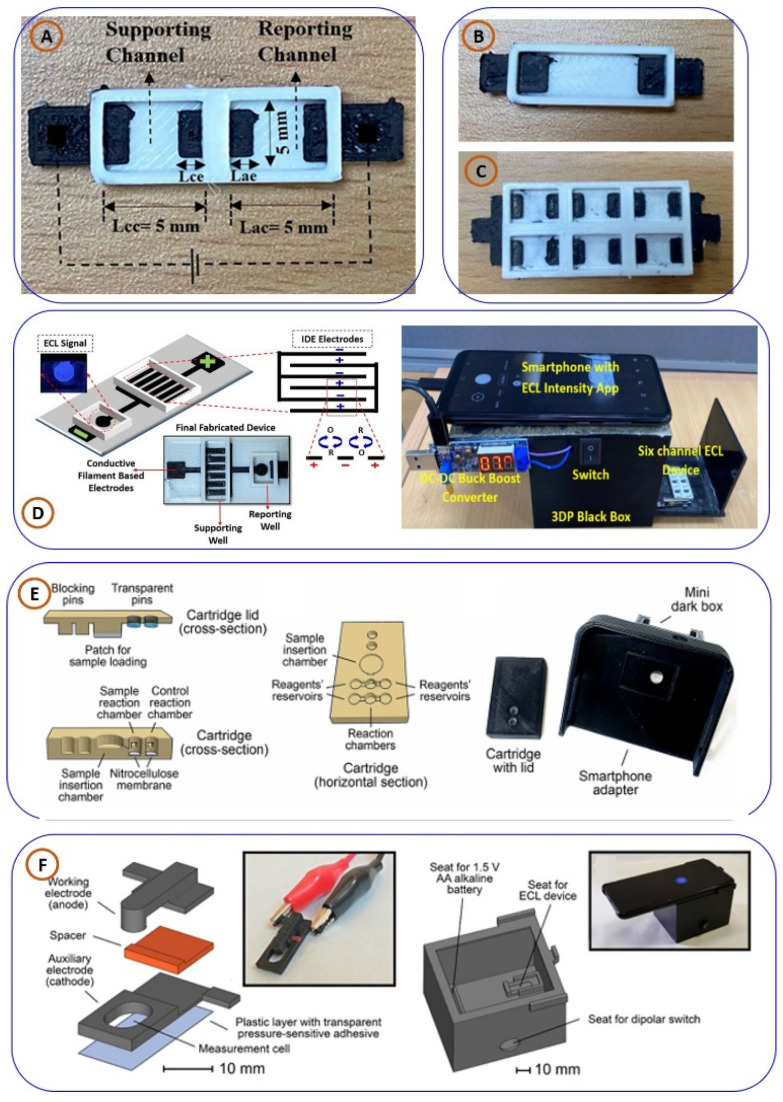
(**A**–**D**) Three-dimensionally printed ECL miniaturized platforms for the detection of various bioanalytes, replicated from [3,8,19,111,112], with the permission of Elsevier; (**E**) chemiluminescence platform for lactate detection, replicated from [113]; (**F**) 3D-printed ECL platforms with portable dark room to detect glucose, replicated from [114].

Vassiliki Katseli et al. [104] developed electrochemical sensing devices produced via 3DP, specifically FDM (Figure 7D). In their study, they demonstrated that 3DP represents a low-cost, adaptable, and sustainable method to fabricate integrated electrochemical platforms. The electrochemical sensing devices were tested to detect caffeine and mercury, and the data reported were comparable to those generated via conventional approaches. In another study, Cristiane Kalinke et al. [25] manufactured immunosensors through 3DP to assist in the diagnosis of Parkinson’s disease. They employed conductive PLA-based filaments to fabricate sensors that could detect the PARK7/DJ-1 protein, a significant biomarker, from blood serum and test samples taken from the central nervous system. Detection was achieved through impedimetric and voltametric approaches, with limits of detection of 1.01 µg/L and 3.46 µg/L for each of the approaches. They established early diagnosis and monitoring approaches for Parkinson’s disease in their innovative approach, with sufficient reliability and reproducibility based on the use of 3DP sensors. To assist in the early detection of liver cancer, Samar Damiati et al. created an inexpensive 3DP biosensor (Figure 8E) targeted to detect the CD133 marker, which is associated with cancer stem cells. Their biosensor combined traditional biosensor components and incorporated a specific protein layer for capturing the target. Detection was achieved in real-time utilizing a quartz crystal microbalance, which indicated promise for future clinical diagnostic applications [105].

In response to the increasing concern regarding mycotoxins in food, Muhammad Zafir Mohamad Nasir and colleagues [106] used a 3DP device (Figure 8F) and demonstrated that it can detect a hazardous mycotoxin known as zearalenone (ZEA). The electrodes used were graphene-based and were fabricated using printing technology and subsequently activated using chemical and electrochemical activation methods. The activated electrodes exhibited high electrochemical activity with a detection sensitivity range of 10 µM to 300 µM, demonstrating applicability for rapid, point-of-care mycotoxin testing. Collectively, these articles continue to demonstrate how 3DP electrochemical sensors are redefining PoCT. The accessibility of quickly designable and manufacturable highly sensitive and adaptable sensor systems represents an emerging approach to medical diagnosis, environmental monitoring, and food safety. To provide a more descriptive comparison, the performance of numerous 3DP electrochemical biosensors is summarized in Table 4; this table describes their target analytes, materials, sensing methods, and statistics.

#### 3.2.2. Three-Dimensionally Printed Electrochemiluminescence (ECL) and Chemiluminescence (CL) Sensors for PoCT Applications

Electrochemiluminescence (ECL) and chemiluminescence (CL) sensors have emerged as powerful tools for PoCT applications due to their high sensitivity, selectivity, and ability to provide rapid results. The integration of 3D printing technology into these sensors has further enhanced their functionality by enabling the fabrication of customized designs for precise, low-cost, and efficient detection platforms.

These advancements have paved the way for the development of portable, smartphone-integrated, and IoT-enabled biosensing systems for real-time diagnostics.

A closed bipolar electrode system capable of choline and dopamine detection based on ECL methods was developed by Dr. Manish Bhaiyya and his team [8] and is depicted in Figure 8A. The system used two ECL chemistries geared towards generating a strong luminescent signal of Ru(bpy)_3_^2+^/TPrA and luminol/H_2_O_2_. The system was further enhanced with smartphone-based imaging with multi-channel photomultiplier tube detection using IoT technology. This configuration enabled remote collection and real-time transmission of data suitable for many of the requirements of modern connected diagnostics. The device demonstrated a good linear response with acceptable limits of detection for both neurotransmitters, highlighting its potential for neurological disorder diagnostics and metabolic monitoring. Expanding upon this work, the same research group designed a six-well 3D-printed ECL platform for the simultaneous detection of glucose and choline, as illustrated in Figure 8C [3]. This innovative system generated six independent ECL signals corresponding to different concentrations of glucose and choline, significantly accelerating the diagnostic process. To enhance usability, the group developed an ECL intensity calculator, which automates tasks such as image capture, signal intensity analysis, and image cropping. Real sample analysis was performed using an additive method, yielding high recovery percentages and confirming the device’s reliability for clinical applications. Further advancements in 3D-printed ECL sensors include the exploration of single-electrode [19,111] and interdigital electrode-based [112] platforms for detecting bioanalytes in human blood serum, as depicted in Figure 8B,D. These systems capitalize on the advantages of miniaturization and enhanced electrochemical activity, providing high sensitivity and reproducibility. By tailoring electrode design and surface modification strategies, these platforms have successfully detected a range of biomolecules with minimal interference from complex biological matrices, making them highly suitable for PoCT devices.

In their contribution to clinical diagnostics, Aldo Roda et al. [113] developed a smartphone-integrated CL biosensing platform for non-invasive lactate detection in sweat and oral fluid (Figure 8E). Utilizing lactate oxidase, the device achieved detection limits of 0.5 mM (sweat) and 0.1 mM (oral fluid), demonstrating strong linearity and suitability for metabolic monitoring in sports medicine and critical care. In another CL-based advancement, Donato Calabria et al. [114] introduced a compact 3D-printed darkroom system paired with a smartphone for glucose detection in serum (Figure 8F). The device employed luminol/H_2_O_2_ chemistry with glucose oxidase and exhibited excellent sensitivity and a consistent linear response. Its successful real-sample application underscores its practicality for home-based glucose monitoring in diabetes care. Extending CL sensing to oncology, Hasan Motaghi et al. [115] developed a 3D-printed closed bipolar electrode system with ECL detection for identifying human breast cancer cells. The platform used AS1411 aptamer-modified gold nanoparticles to selectively bind nucleolin, an overexpressed cancer biomarker, yielding high sensitivity and specificity. This low-cost, real-time biosensor highlights the growing potential of 3D-printed ECL platforms in personalized cancer diagnostics and biomarker monitoring.

### 3.3. Three-Dimensionally Printed Lab-on-Chip (LoC) and Microfluidic Devices for PoCT Applications

The utilization of lab-on-chip and microfluidic devices represents some of the most technologically advanced and influential forms of 3D printing in healthcare. Lab-on-chip and microfluidic devices comprise a collection of laboratory tests and processes consolidated onto a microchip that allow for fast and efficient laboratory analysis of biological samples. With 3DP and its precision advantages, it has been established that complex microfluidic channels, valves, and sensors are now possible, and, consequently, associated devices can be engineered to satisfy a personalized diagnostic situation. The studies that follow support how 3D printing is changing design and use paradigms of LoC and microfluidic platforms that are innovating biosensing, chemical analysis, and real-time health calibrators.

**Figure 9:**
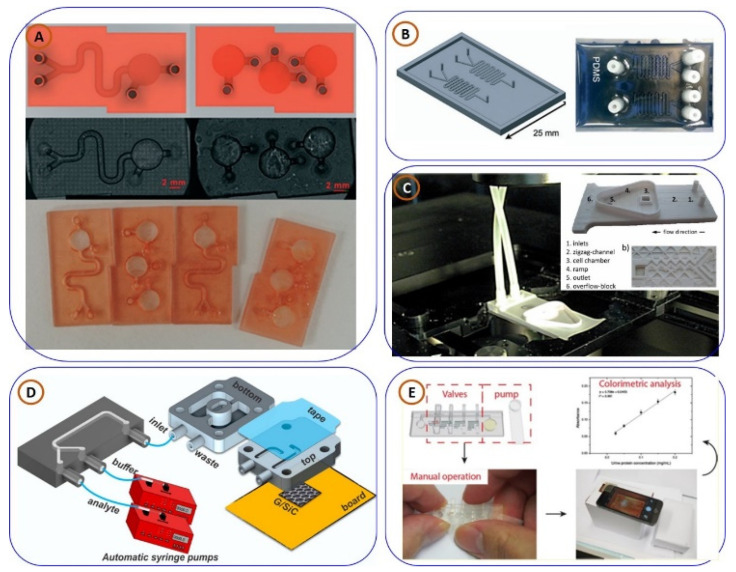
Various 3D-printed lab-on-a-chip (3DP-LoC) platforms designed for biomedical and diagnostic applications: (**A**) a 3DP-LoC device for detecting endothelial growth factor and angiopoietin-2 biomarkers, taken from [116], copyright RSC; (**B**) a microfluidic device created through 3D printing for glucose detection, taken from [58], copyright RSC; (**C**) a 3DP-LoC setup designed for fluorescence microscopy-based calcium imaging, taken from [117], copyright MDPI; (**D**) a 3DP chemical sensor for PoCT application setup, taken from [118], copyright MDPI; (**E**) a 3DP-LoC microfluidic chip used for protein quantification, taken from [119], copyright ACS.

Alessandro Chiadò et al. [116] pioneered the use of SLA-based 3D printing for lab-on-chip applications, developing a modular device for the detection of biomarkers such as endothelial growth factor and angiopoietin-2 (Figure 9A). Targeting angiopoietin-2, an important biomarker for early breast cancer, the device achieved a remarkable detection limit of 0.8 ng/mL, well below the diagnostic threshold. To promote affordability and accessibility in lab-on-chip fabrication, Germán Cominas et al. [58] designed a PDMS-on-glass system using a micro-stereolithography 3D printer (Figure 9B). Their approach eliminated the need for cleanroom environments and reduced fabrication costs, while successfully enabling glucose detection, making it suitable for scalable chemical sensing applications. In the domain of functional imaging, Daniel Aschenbrenner et al. [117] developed an ultra-low-cost microfluidic lab-on-chip platform using ABS and 3D printing (Figure 9C). Designed for in vitro imaging of ion channels, the device facilitated precise solution exchange and calcium imaging via fluorescence microscopy. The study also confirmed the biocompatibility of ABS, validating its suitability for biological applications and its versatility for analyzing ion channel activity.

Maria Francesca Santangelo et al. [118] explored the integration of nanomaterials into lab-on-chip platforms by employing graphene as a transducer material for chemical sensing (Figure 9D). Their device demonstrated improved detection limits and miniaturization, particularly in detecting heavy metals like cadmium and lead. A novel configuration combining epitaxial graphene with 3D-printed microfluidics was introduced, highlighting its suitability for real-world environmental and field applications. Advancing microfluidic chip functionality, Ho Nam Chan et al. [119] developed mechanically tunable 3D-printed chips incorporating rotary and pushing valves along with a torque-actuated pump (Figure 9E). These features eliminate the need for external pressure sources, enabling compact and portable diagnostic solutions. The system was validated by performing protein quantification in synthetic urine using a smartphone-based imaging setup, showcasing its practicality in point-of-care diagnostics.

**Figure 10:**
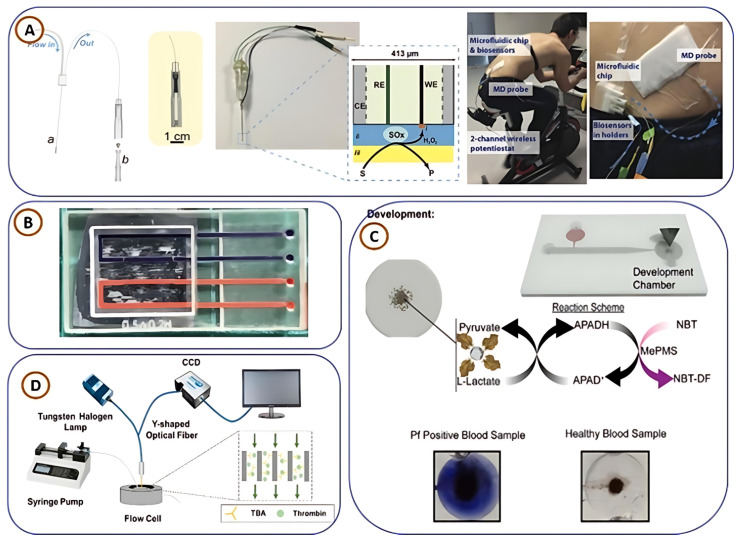
(**A**) Three-dimensionally printed microfluidic analysis system for continuous monitoring of human tissue metabolite levels, replicated from [88], copyright ACS. (**B**) Polyacrylate-based 3DP microfluidic aptasensor platform, replicated from [120], copyright springer. (**C**) Three-dimensionally printed portable microfluidic biosensor for malaria diagnosis, replicated from [121], copyright Elsevier. (**D**) Three-dimensionally printed rapid and reagentless detection of thrombin microfluidic platform, replicated from [122], copyright Elsevier.

In a shift toward continuous real-time monitoring, Gowers et al. [88] designed a 3D-printed microfluidic system compatible with FDA-approved microdialysis probes (Figure 10A). The platform integrates needle-type biosensors to monitor glucose and lactate levels in subcutaneous tissue. Successfully demonstrated during a cycling trial, the device supports wearable metabolic monitoring and holds significant translational and clinical potential due to its modularity and commercial compatibility. For microbial sensing, Siller et al. [120] developed 3D-printed flow cells equipped with static/dynamic chambers and micro-mixers to detect *E. coli* Crooks strain (Figure 10B). The device utilized aptamer-functionalized gold screen-printed electrodes (SPEs) and electrochemical impedance spectroscopy (EIS) to accurately quantify bacterial concentrations, underscoring its relevance in food safety and pathogen detection.

In another study, pushing the boundaries of biosensing technologies, Sofia Arshavsky-Graham et al. [121] designed a polyacrylate-based microfluidic aptasensor platform, shown in Figure 10C. The device was tested for label-free identification of His-tagged proteins, comparing results with conventional non-microfluidic and PDMS-based sensing systems. Their findings revealed that the 3D-printed microfluidic aptasensor exhibited superior sensitivity, selectivity, and detection limits, proving its effectiveness in next-generation biosensing applications. In continuation, focusing on disease diagnostics, Lewis A. Fraser et al. [122] presented a portable microfluidic biosensor for malaria diagnosis, shown in Figure 10D. This 3D-printed device, combined with magnetic beads and a smartphone-based colorimetric detection system, successfully identified Plasmodium falciparum lactate dehydrogenase, a key malaria biomarker. The study demonstrated high specificity and sensitivity, highlighting its potential for rapid, on-site malaria diagnostics using clinical samples. Finally, concluding with advancements in blood coagulation analysis, Neng Yu et al. [123] developed a microfluidic aptasensor for rapid, reagent-free thrombin detection. Utilizing porous silicon membranes functionalized with thrombin-binding aptamers (TBAs), the biosensor enabled real-time detection through reflective interferometric Fourier transform spectroscopy. The study achieved detection limits of ~6.70 nM in buffer and ~8.21 nM in serum, demonstrating its suitability for blood clot-related diagnostics. Additionally, the biosensor’s rapid regeneration using a high-salt elution process ensures its reusability and cost-effectiveness.

The case studies presented above illustrate the transformative potential of 3D printing in the development of lab-on-chip and microfluidic devices, showcasing a wide range of applications in healthcare, from disease diagnostics to real-time health monitoring. These innovations highlight the versatility of 3DP in fabricating complex microfluidic structures, integrating advanced sensing mechanisms, and enabling cost-effective, portable solutions for point-of-care testing. The following comparative Table 5 provides a detailed overview of these 3DP lab-on-chip devices, summarizing their applications, materials, sensing mechanisms, key features, and statistical analyses. This table serves as a comprehensive reference to understand the advancements and practical implications of 3D printing in microfluidics and biosensing.

## 4. Challenges and Future Scope

While 3DP fabrication methodology for PoCT devices represents an innovative advancement, it encounters several challenges in the global market, a few of which are discussed here.

### 4.1. Challenges in 3DP PoCT Devices

#### 4.1.1. Standardization

The lack of standardized protocols or guidelines for the design, fabrication, and quality control of 3DP-PoCT devices is a significant barrier to their widespread adoption. Consistency and reliability in diagnostic results are essential for clinical applications, but without standardized processes, there is a risk of variability in device performance. This variability can lead to unreliable diagnostic outcomes, hindering trust in 3DP-PoCT devices among healthcare providers and patients. Standardization is necessary to ensure that devices meet uniform quality and performance benchmarks, regardless of where or how they are manufactured. Developing industry-wide standards for 3D printing processes, material selection, and device validation will be crucial. Collaboration between regulatory bodies, researchers, and manufacturers can help establish these protocols [11,129].

#### 4.1.2. Material Selection

The selection of suitable materials for 3DP-PoCT is riddled with challenges. These materials are expected to meet multiple conflicting requirements: biocompatibility, that is, having no negative effect on the patient; mechanical stability, to withstand stress during operation; and ease of processing, enabling precise fabrication through various 3D printing technologies. Inadequate material properties may lead to problems with device functionality, safety, and durability. For instance, materials that are not biocompatible can cause adverse reactions in patients, whereas those with low mechanical strength may fail during use. Continuous research into modern materials, such as biocompatible polymers, nanocomposites, and conductive materials, will provide substantial information on this subject. Therefore, specific applications of these materials, such as biosensing and drug delivery, may become more effective and reliable [18,21].

#### 4.1.3. Regulatory Compliance

Examples of non-satisfactory or faulty print materials, quality concerns, regulatory non-compliance, low user standards, limited applications, and unpursued use in medical contexts are very few, which is positive regarding the flexibility and strength of 3D-printed technologies under stress conditions. Regulatory protocols, on the other hand, often vary between the multiple regions of distribution and introduce further complications for manufacturers targeting a global reach for their solutions. The consequences of non-compliance with regulations can be serious, and they can bring about delays in the approval of devices, restrict access to the market, and put public confidence in 3DP-PoCT technologies at risk. Hence, meeting all the regulatory standards is imperative for their consideration in a true clinical setting. It is obligatory for manufacturers to reach out to the regulatory agencies for consultation to address their apprehensions about safety and efficacy. Only by putting a lot of work into quality control do they stand a chance of adequately conducting clinical trials to demonstrate compliance and instill confidence in this technology in their fellow humans [130].

### 4.2. Future Opportunities for 3DP-PoCT Devices

#### 4.2.1. Advanced Materials

The new generation of materials from bioengineering and nanotechnology seems likely to offer enhanced features, such as advanced biocompatibility, increased mechanical strength, and higher electrical conductivity, for the next generation of 3DP-PoCT devices. Such tailored materials will enable the design of better-fitted devices for specific diagnostic or therapeutic applications, thus advancing their accuracy, sensitivity, and patients’ well-being. Conductive graphene-based materials would enhance biosensing capabilities, while biodegradable polymers would allow environmentally friendly single-use devices to be developed [131].

#### 4.2.2. Multifunctional Devices

In the future, 3DP-PoCT devices might integrate different functionalities, such as biosensing, drug delivery, imaging, and real-time monitoring, in one platform. Such integration could be made possible largely due to the advancement of microfluidics, electronics, and IoT technologies. Multifunctional devices will provide more integrated healthcare solutions, which means they will perform diagnostic, treatment, and monitoring functions concurrently. This is expected to make healthcare delivery more efficient, lowering its costs and improving patient outcomes. A single device could then detect a biomarker, deliver a targeted drug, and monitor a patient’s response to that drug in real time, while streaming the data back to a healthcare provider thanks to IoT connectivity [12,132].

#### 4.2.3. Integration of Emerging Technologies

**Figure 11:**
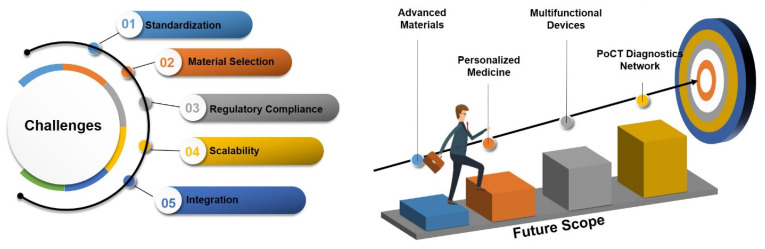
Diagrammatic representation of challenges associated with 3DP-PoCT devices and future scope for exploring opportunities.

The inclusion of AI, ML, and IoT within 3DP-PoCT devices will change the game for diagnosis and monitoring in the health sector. AI and ML will analyze complex data from biosensors to enhance diagnostic accuracy, while IoT allows data sharing and remote monitoring in real time. These technologies will boost enhanced 3DP-PoCT device functioning, making them more intelligent, efficient, and accessible. They will also enable personalized medicine by tailoring diagnostics and treatments to individual patients. An AI-powered 3DP-PoCT device could analyze a patient’s blood sample, predict the risk of disease, and recommend personalized treatment approach—all within minutes [36,37,39]. Figure 11 shows a diagrammatic representation of the challenges associated with 3DP-PoCT devices and the future scope for exploring opportunities. There are certain immense challenges to the standardization of 3DP-PoCT devices that mainly relate to material selection and regulatory compliance; however, 3D printing offers great promise to reform healthcare. After addressing these challenges, taking advantage of advances in material science, multifunctional integration, and the incorporation of new technologies such as AI and IoT, economically exploitable 3D printing-based diagnostics, therapeutics, and therapy monitoring devices might be healthcare’s future. Moving ahead, 3DP-PoCT devices need to overcome current limitations and address opportunities for emerging innovative, accessible, and impactful solutions for healthcare. Continued research and collaboration will certainly make these devices important contributors to healthcare delivery and improved patient outcomes on a global scale.

## 5. Conclusions

Three-dimensional printing technology has heralded a new direction in the diagnostics industry, transforming point-of-care testing, biosensing, wearable health monitoring devices, and implantable medical solutions. Very small, inexpensive, and highly customized medical devices could be manufactured through additive manufacturing and address the challenges of traditional centralized laboratory diagnostics, such as high costs, years of turnaround, and constrained accessibility in resource-scarce regions. Integration with biosensors, microfluidic lab-on-chip systems, and smart wearable technologies through 3D printing enables real-time health monitoring and personalized medicine, further improving patient outcomes and decentralized healthcare. Despite the anticipated transformation that will come with this technology, there are still challenges related to material standardization, regulatory compliance, and scalability, requiring continued research and synergy between academia, industry, and regulatory agencies. Therefore, future developments in multifunctional PoCT devices and biocompatible materials and the convergence of 3DP with artificial intelligence and IoT would further their economic diagnostic performance, efficiency, and access. Addressing such challenges by adopting technological innovations will ensure that 3DP-driven PoCT solutions continue to transform the face of healthcare, such that people have equitable access to a high standard of diagnostic tools and personalized treatment. The continuous evolution of 3DP in healthcare promises to create a more patient-centered, cost-effective, and technologically advanced medical ecosystem.

## Figures and Tables

**Table 1:** Comparison of 3D printing techniques for PoCT applications [1,2,36,37,39,60,61].

Attribute	FDM	SLA	PBF	BJ	DED	LOM
Material Used	PLA, ABS, PETG	Photopolymers	Metals, polymers	Metal, ceramic powders	Metals	Paper, plastic, metal
Resolution (µm)	100–300	<50	<100	100–200	<50	200–400
Speed (mm/s)	50–150	10–100	20–80	50–200	5–20	50–100
Energy Consumption (W)	50–150	100–250	200–500	150–300	500–1000	30–80
Surface Roughness (Ra, µm)	10–25	1–5	5–15	15–30	5–20	25–50
Tensile Strength (MPa)	20–50	40–65	100–150 (metal)	20–40	150–300	15–30
Typical Applications	Prototypes, medical models, educational aids	Dental models, hearing aids, jewelry	Functional parts, implants, aerospace	Molds, architectural models	Repairs, implants, aerospace components	Packaging, low-cost prototypes
Drawbacks	Lower precision, poor surface quality	Resin handling issues, material limitations	Expensive, material-specific, needs inert gas	Weak mechanical strength, post-processing required	High equipment cost, complex process control	Low bonding strength, poor resolution
Estimated Cost per Device (USD)	1–5	3–10	10–100	5–15	20–150	1–3

**Table 2:** Material properties and their impact on 3D-printed PoCT devices.

Material Type	Key Properties	Impact on PoCT Devices
Graphene–PLA Composites	High conductivity, biocompatibility	Enhanced electrochemical sensing capabilities
Photopolymer Resins	High resolution, optical clarity	Precise microfluidic channel fabrication
PDMS	Flexibility, optical transparency	Suitable for microfluidic devices and wearable sensors
SEBS	Elasticity, skin compatibility	Ideal for stretchable wearable sensors
Hydrogels	High water content, biocompatibility	Mimic biological tissues for biosensing applications
MXenes	Electrical conductivity, flexibility	Development of sensitive and flexible wearable biosensors
MOFs	High surface area, porosity	Improved sensitivity and selectivity in analyte detection

**Table 3:** Comparison of 3D-printed wearable sensors.

Ref. No.	Application	3D Printing Material	Sensing Mechanism	Key Features	Statistical Analysis
[72]	Stretchable and Wearable Sensors	Multi-Walled Carbon Nanotubes (MWNT) and PDMS	Piezoresistive Sensing	Highly flexible, strain detection up to 146%	Sensitivity (gauge factor = 12.15), tested under cyclic loads
[74]	High-Precision Wearable Biosensors	3D-Printed Sugar Scaffold	Capacitive and Resistive	Personalized, lightweight, and highly sensitive	Statistical validation for EMG, EDA, and EEG sensing
[73]	Stretchable Tactile Sensors	Conductive Polymer Composite	Piezoresistive	High sensitivity, used for prosthetics	Response time and mechanical stability validated
[75]	Ionic Skin Sensors	Photo-Polymerized Hydrogel	Ionic Conductivity	Skin-like elasticity, high-resolution 3D printing	Linearity and LOD analysis provided
[76]	Bioelectronic Sweat Monitoring Patch	3D-Printed Flexible Sensors	Electrochemical	Detects multiple electrolytes in sweat	Real-time health monitoring, reproducibility tested
[79]	Piezoresistive Health Monitoring Sensor	CNT Surface-Filled SEBS Substrate	Piezoresistive	Highly stretchable, stable	2000+ cycle durability and response time (149 ms) measured
[82]	Wearable Smart Device	Liquid Metal and 3D-Printed Polymer	Infrared and Acoustic	Core body temperature and bone conduction	Real-time data accuracy comparison with clinical tools
[83]	Personalized Oral Drug Delivery	PLA-PVA 3D-Printed Device	Drug-Release Mechanism	Custom-fit mouthguard with tunable release	First-in-human study, drug diffusion kinetics analyzed
[84]	Recyclable Wearable Electronics	Dynamic Thermoset Elastomer	Capacitive and Triboelectric	Fully degradable and recyclable electronics	Mechanical durability and recyclability efficiency evaluated
[85]	Respiratory and Heart Rate Monitoring	3D-Printed FBG-Based Sensor	Optical Strain Sensing	High accuracy HR and RR monitoring	Metrological properties validated
[86]	Skin-Like Wearable Strain Sensors	Self-Healable Hydrogel with CNTs	Piezoresistive and Piezoelectric	Multifunctional, real-time response	Sensitivity: GF = 6.29 (resistance), 1.25 kPa^−1^ (capacitance)
[87]	Ultra-Robust Biomonitoring Sensors	Graphene-Doped Porous Silicone	Piezoresistive	Long-term durability (>12 months), stable under 75% compression	400+ cycle durability, resistance stability validated
[88]	Microfluidic Biosensor for Human Tissue	3D-Printed Electrode Holders	Electrochemical	Continuous glucose and lactate monitoring	Real-time data validation on cyclists
[89]	Smart Fibers and Textiles	MXene-Reinforced Cellulose Nanofibrils	Electrical, Mechanical, Photonic	High flexibility, responsive to multiple stimuli	Wearable heating and sensing applications tested
[90]	Stretchable Thermoelectric Generators	PEDOT:PSS Composite	Thermoelectric	Self-healing, maintains > 85% power after damage	Power output: 12.2 nW, retained post-cutting

**Table 4:** Comparison of 3D-printed electrochemical biosensors.

Ref. No.	Detection Target	3D Printing Material	Electrochemical Method	Sensitivity and Detection Limit	Statistical Analysis
[96]	Hepatic Oval Cells (HOCs)	MWCNTs with Chitosan Film	Cyclic Voltammetry and Square-Wave Voltammetry	Enhanced sensitivity due to MWCNT scaffold	Reproducibility tested; RSD values provided
[97]	Insulin and ATP	Epoxy-embedded electrodes with microfluidic devices	Aptamer-based electrochemical detection	Simultaneous detection under flow conditions	Linearity and LOD values evaluated
[98]	Cardiac Biomarkers (Cholesterol and Choline)	Enzymatic 3D-printed microchip	Amperometric determination	Low LOD for cardiac biomarkers (3.36 and 0.08 μm)	Multiplexed assay statistical validation
[99]	Hydrogen Peroxide (H_2_O_2_)	Conductive graphene filaments	Direct Electron Transfer (DET)	No need for mediators, stable response: LOD 11.1 and 9.1 μM for H_2_O_2_	Repeatability tests and comparative performance with traditional methods
[25]	Parkinson’s Disease Biomarker (PARK7/DJ-1 Protein)	PLA-based conductive filaments	Impedimetric and Voltammetric Analysis	LOD: 1.01 µg/L (impedimetric), 3.46 µg/L (voltammetric)	Repeatability and reproducibility confirmed
[103]	SARS-CoV-2 cDNA and Creatinine	AuPs-modified graphene–PLA electrodes	Square-Wave Voltammetry	LOD for SARS-CoV-2 cDNA: 0.30 µmol/L	RSD = 1.14%, n = 3
[100]	Hydrogen Peroxide and Glucose	Silver microelectrode arrays	Amperometric sensing	LOD: 0.45 µM (H_2_O_2_), 1.7 µM (glucose)	Sensitivity analysis and diffusion limitations tested
[107]	Cadmium and Lead in Biological Fluids	Carbon black–PLA electrodes	Square-Wave Anodic Stripping Voltammetry (SWASV)	LOD: 2.9 µg/L (Cd^2+^), 2.6 µg/L (Pb^2+^)	RSD < 6.5%, high reproducibility
[105]	Liver Cancer Cells (HepG2)	Hybrid 3D-printed electrochemical sensor	Cyclic Voltammetry and Quartz Crystal Microbalance (QCM-D)	Highly selective for CD133 biomarker	Real-time detection with label-free analysis
[108]	Aflatoxin B1 (AFB1)	3D-bio-printed liver lobule microtissue	Differential Pulse Voltammetry	LOD: 0.039 µg/mL	Stability and reproducibility tested
[109]	Blood Urea	Gold nanoparticle-integrated 3D-printed chip	Linear Sweep Voltammetry	LOD: 0.1 µM, Sensitivity: 183 µA mM^−1^ cm^−2^	RSD = 3.63%, shelf life > 6 months
[110]	Steroid Hormones (Estradiol and Progesterone)	PLA-CB and ABS electrochemical cell	Differential Pulse Voltammetry	LOD: 0.11 µmol/L (E2), 17.8 µmol/L (P4)	RSD = 3.1% (repeatability), 10.7% (reproducibility)
[102]	Escherichia coli	3D-printed graphite pencil electrode	Cyclic Voltammetry	LOD: 53 CFU/mL, LOQ: 270 CFU/mL	Cost-effective and rapid detection (USD 2.50/test)
[104]	Multipurpose Electrochemical Sensing	Carbon-loaded PLA electrodes	Single-Step 3D Printing	Versatile detection with a broad potential range	High precision and reproducibility

**Table 5:** Comparison of 3D-printed lab-on-a-chip devices.

Ref. No.	Application	3DP Material	Sensing Mechanism	Key Features	Statistical Analysis
[124]	Biodegradable Drug Delivery Implants	PLA and PCL	Controlled Drug Release	Personalized, long-duration drug release	Drug diffusion kinetics analyzed
[125]	Malaria Detection	FDM 3D-Printed Fluidic Cartridge	Colorimetric ELISA	Portable, automated reagent dispensing	Cost-effective with smartphone-based analysis
[117]	Ion Channel Functional Analysis	ABS Microfluidic Chip	Fluorescence-Based Functional Imaging	Low-cost, reproducible, high throughput	Homogeneity of solution exchange validated
[118]	Heavy Metal Detection (Pb and Cd)	Epitaxial Graphene	Conductometric	Fast response, real-time detection	Sensitivity (13.90 Ω/µM) tested with Langmuir isotherm correlation
[119]	Urinary Protein Quantification	3DP Microfluidic Components	Colorimetric Analysis	Simple, cost-effective, portable	Smartphone-based quantification tested (LoD: 8.5 μg/mL)
[88]	Online Subcutaneous Monitoring	Integrated Microfluidic Biosensors	Electrochemical Detection	Real-time glucose (6.02 ± 1.08 mM) and lactate (1.81 ± 0.33 mM) monitoring	Wireless connectivity tested in athletes
[120]	E. coli Detection	3DP Flow Cells	Impedimetric Aptasensor	High specificity, microfluidic integration	Sensitivity analysis performed
[121]	Protein Detection	Polyacrylate-Based Microfluidic Platform	Optical Aptasensor	High selectivity, improved LOD	Comparative performance with PDMS microfluidics
[122]	Malaria Diagnosis	3DP Microfluidic Chambers	Aptamer-Tethered Enzyme Capture (APTEC)	Portable, high sensitivity (90% across all patient samples)	Clinical sample validation conducted
[123]	Thrombin Detection	Open-Ended Porous Silicon	Reflective Interferometric Fourier Transform Spectroscopy (RIFTS)	Rapid, reagentless detection	ELISA-based verification tested (LoD: ∼6.70 nM)
[98]	Multiplexed Cardiac Biomarker Detection	3DP Enzymatic Microchip	Amperometric Electrochemical Biosensing	Simultaneous cholesterol and choline detection	LOD 3.36 and 0.08 μm
[116]	Modular Early Cancer Detection	Functional Polymeric 3D Device	Immunoassay for Protein Biomarkers	Rapid, cost-effective, scalable	LOD for VEGF: 11 ng/mL, Angiopoietin-2: 0.8 ng/mL
[126]	DIY ELISA Plate Reader	3DP Optical Sensor	Colorimetric Detection	Low-cost alternative to commercial plate readers	LOD: 19 pg/mL for TNFα assay
[127]	Glucose, Uric Acid, and Nitrite Detection	Graphene–PLA Electrodes	Differential Pulse Voltammetry	Multi-analyte sensing in biological fluids	LOD: 0.02 µM (uric acid), 0.03 µM (Nitrite), 15 µM (glucose)
[128]	Tetracycline Antibiotic Detection	Conductive Graphite–PLA Electrode	Amperometric BIA-AD System	High sensitivity, food and water safety application	LOD: 0.19 µM, recovery: 92–117%
